# Gene Set Based Integrated Data Analysis Reveals Phenotypic Differences in a Brain Cancer Model

**DOI:** 10.1371/journal.pone.0068288

**Published:** 2013-07-09

**Authors:** Kjell Petersen, Uros Rajcevic, Siti Aminah Abdul Rahim, Inge Jonassen, Karl-Henning Kalland, Connie R. Jimenez, Rolf Bjerkvig, Simone P. Niclou

**Affiliations:** 1 Computational Biology Unit, Uni Computing, Uni Research AS, Bergen, Norway; 2 Norlux Neuro-Oncology Laboratory, Department of Oncology, Centre de Recherche Public Santé, Luxembourg, Luxembourg; 3 Blood Transfusion Centre of Slovenia, Ljubljana, Slovenia; 4 Department of Informatics, University of Bergen, Bergen, Norway; 5 The Gade Institute, University of Bergen, Bergen, Norway; 6 Department of Pathology, Haukeland University Hospital, Bergen, Norway; 7 OncoProteomics Laboratory, Department of Medical Oncology, Vrije Universiteit (VU) Medical Cancer Center, Amsterdam, The Netherlands; 8 Norlux Neuro-Oncology, Department of Biomedicine, University of Bergen, Norway; University of Georgia, United States of America

## Abstract

A key challenge in the data analysis of biological high-throughput experiments is to handle the often low number of samples in the experiments compared to the number of biomolecules that are simultaneously measured. Combining experimental data using independent technologies to illuminate the same biological trends, as well as complementing each other in a larger perspective, is one natural way to overcome this challenge. In this work we investigated if integrating proteomics and transcriptomics data from a brain cancer animal model using gene set based analysis methodology, could enhance the biological interpretation of the data relative to more traditional analysis of the two datasets individually. The brain cancer model used is based on serial passaging of transplanted human brain tumor material (glioblastoma - GBM) through several generations in rats. These serial transplantations lead over time to genotypic and phenotypic changes in the tumors and represent a medically relevant model with a rare access to samples and where consequent analyses of individual datasets have revealed relatively few significant findings on their own. We found that the integrated analysis both performed better in terms of significance measure of its findings compared to individual analyses, as well as providing independent verification of the individual results. Thus a better context for overall biological interpretation of the data can be achieved.

## Introduction

The rapid progress in technology development for assessing information from multiple angles about genes, proteins and metabolites, has resulted in a growing expectation of a large potential for new discoveries in the understanding of cellular molecular activities. Individual monitoring technologies have been marketed to reveal a holistic picture by capturing information about most entities of a type, as for instance all transcribed genes encoded in the genome or a large number of proteins present in a prepared sample. Obviously, a natural extension is the combination of several types of data to reveal more information about biological processes at the molecular level. To reap from this expected potential of discoveries, several fundamental challenges have to be faced. High throughput datasets have by nature a large imbalance between number of samplings and number of variables measured, leading to challenges regarding interpretation and confidence estimates of analysis results. And the interpretation of several datasets assessing samples from different angles in combination requires a new theoretical model which can assess biological questions and significance of predicted answers. A successful integrated model should assess relevant biological questions with higher confidence in predicted answers compared to methods for individual dataset types, despite the increased complexity of the model. In this work we present a combined analysis approach for interpreting high throughput microarray and proteomics datasets on two different tumor phenotypes obtained by serial transplantations of human GBMs in the CNS of rats [1], [2].

**Figure 1:**
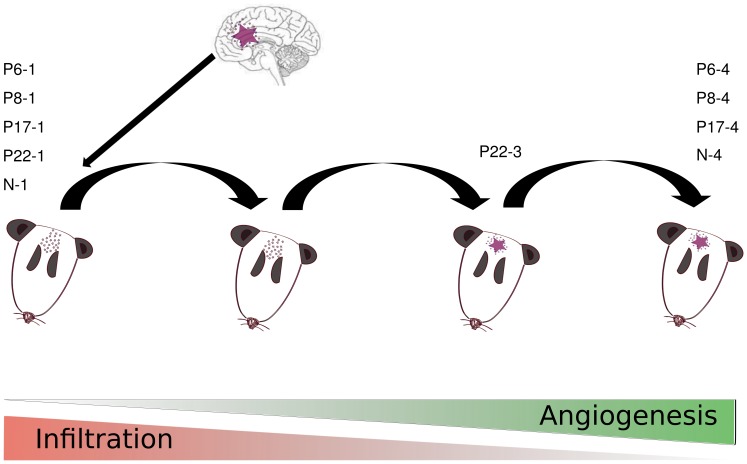
Orthotopic Xenograft Brain Tumor Model. A schematic representation of the tumor model and the phenotypes obtained after transplantation in nude rats. The first transplantation into nude rats often resulted in an invasive phenotype, while serial transplantation of the tumors resulted in angiogenic phenotype after several generations.

GBM represents a heterogeneous group of malignant brain tumors [3] and is one of the most fatal forms of cancers in humans. The average survival of affected patients has only improved from an average of 12 months to 14.5 months after diagnosis in the last 5 years due to improvements in standard of care [4]. To address the complex issue on the molecular background of human GBMs, a human GBM model was developed in immunodeficient rats [1], [2], [5], which partially uncouples two major phenotypic characteristics and landmarks of this tumor, *i.e.* invasion and angiogenesis. These two characteristics render GBM difficult to treat by available therapies. The model is based on serial xenotransplantation of human GBM spheroids into the brain of immunodeficient rats, where they initiate the growth of primary GBMs. The phenotype of the first generation tumor shows a highly invasive nature in the rat brain whereas by serial passaging in the animals, the tumor evolves into a faster growing angiogenic tumor, with abundant vasculature, and less invasion. The model and brain tissue phenotypes are illustrated in Figure 1.

As already mentioned, data analysis and biological interpretation of high-throughput technology generated data sets at the scale of genomes and proteomes is in general a challenge, due to the large imbalance between the number of samples and the number of molecules being tested. To identify a statistical significant change in expression level for a single gene at the level of change that is interesting for biological interpretation, many independent replicates are required in the experiment. The intricate nature of the GBM xenotransplantation serial passage rat model, and the naturally limited availability of tumor material donors, have resulted in a limited set of matched sample pairs with the invasive and angiogenic phenotype to be screened by microarrays and proteomics. In addition, a high level of individual variance between samples is expected and has been observed when addressing the transcriptomics data set in earlier work [1], [6]. The molecular background of the phenotype switch was addressed at the levels of differential expression of RNA [1] and proteins [7]–[9], where extensive validation including large numbers of GBM patients and functional analyses led to novel candidate biomarkers of a particular phenotype [7]–[9]. The challenge however remains to pinpoint particular molecular pathways reflected by enrichment of particular gene sets, which would lead to a better biological understanding of the underlying pathology.

Two general strategies to counter weigh the dimensionality challenges of high-throughput data analysis are (i) to analyze sets of *a priori* defined biologically related molecules at the time instead of individual molecules and (ii) to integrate results from several independent analyses possibly from different high-throughput experiments, both to find evidence supporting the same biological trends and to complement each other for a richer interpretation. The common analysis of Gene Ontology terms overrepresented in a list of differentially expressed genes compared to the full data set is an early example of strategy (i), while the Gene Set Enrichment Analysis – GSEA [10] and the large number of variants of enrichment based methods [11], [12] represents later developments. Several methods of meta-analysis of independent experiments on the same samples exist, from simple Rank Product based combining of individual list results [13] to more complex multi-variate analysis based methods to identify similar trends across the data sets such as Co-Inertia Analysis (CIA) [14], [15]. Multi-variate analysis methods require a minimum number of samples in a dataset, and CIA requires the exact same samples to be present across the datasets, often making them unsuitable in practice, such as in our GBM case. Subramanian et al demonstrated the flexibility of GSEA as a tool for co-analyzing several independent micorarray experiments on biologically related samples. Here we extend this line of thought to cross the barrier between different high throughput technologies.

In this work we applied the Gene Set analysis approach to co-interpret the two datasets in the context of each other. The annotation of the identified genes and proteins are interpreted with respect to the invasive and angiogenic phenotypes, and compared with the regular Gene Ontology analysis results of the individual datasets. This approach highlights how they support and strengthen each other in our combined interpretation, as well as complement each other in a better detailed picture of the phenotypic differences in the brain cancer model’s invasive and angiogenic phases. The results display a strong statistical support between the proteomics and microarray results, which also is reflected in the biological interpretation of the data through a high concordance with the individual analysis results. To further demonstrate the validity of the suggested approach, the results are contrasted with Rank Product meta-analysis of the same two datasets. We also applied the method to an earlier published independent pair of microarray and proteomics data sets, successfully rediscovering the main findings from the original publication.

## Materials and Methods

### Experiment Design

Five pairs of corresponding invasive and angiogenic samples from the xenograft models, originating from five individual patients, were used in total in the microarray and proteomics experiments. Four sample pairs were prepared for microarray analysis and were hybridized to eight Applied Biosystems Human Genome Survey Microarrays v.2.0 (Array Express accession A-MEXP-503) in one hybridization run, as described in [6]. Two sample pairs were prepared for proteomics analysis and processed in three iTRAQ experiments as described in [9]. One sample pair overlapped between the two technologies.

### Preprocessing and Normalization

The microarray data were imported into the data analysis suite J-Express 2012 [16] (http://jexpress.bioinfo.no), for preprocessing and normalization. The raw signal intensities were extracted, controls filtered out, and the data quantile normalized [17]. Further the data were log2 transformed and each sample pair was combined to a single log-ratio column. The proteomics data were preprocessed from raw data to quantified peptides as described in [9], including annotation on origin of peptide from either host cells, tumor cells or unknown origin, based on sequence homology to rat and human databases. In this work we use the full proteomics dataset of 3359 protein profiles.

### Differential Expression Statistics

The Rank Product (RP) statistics [13] was used both for the transcriptomics and proteomics data sets to rank genes and proteins according to differential expression between the invasive and angiogenic samples. RP was also used on the reduced datasets containing only the uniquely mapping transcripts and proteins used for the integrated analysis of the data from the two technologies. RP was implemented in the J-Express 2012 analysis suite.

### Gene Ontology Over-representation Analysis

J-Express uses a Fischer’s exact test to assess statistical overrepresentation of genes annotated with a given Gene Ontology (GO) term (www.geneontology.org, [18]) in a smaller list of interest compared to a reference data set. In this work we compared the top lists of the RP differential expression analysis at a given significance level (q-value) against the full dataset the RP analysis was performed on. Listed p-values for the GO terms in the result table are nominal, *i.e.* not adjusted for multiple testing, and should be evaluated with this in mind. Gene Ontology OBO file used was dated 2010 Dec 3rd, filtered Homo sapiens Gene Ontology mapping file used was dated 2011 Nov 29th. Only GO terms present in the OBO file are included in the analysis.

### Gene Set Enrichment Analysis

As an alternative to the GO overrepresentation analysis, the Gene Set Enrichment Analysis (GSEA) [10] was also applied to evaluate and rank GO terms annotating the two datasets. In contrast to the over-representation analysis, GSEA and related approaches do not operate with a fixed limited list of interest to evaluate. Instead they evaluate the distribution of genes annotated with a given GO term across the reference data set. In GSEA the distribution is used to define a natural subset of the annotated genes called the Leading Edge (LE) that contributes to the score of the gene set (GO term in this case), and that can be followed up for a closer biological interpretation. The analyses were performed with the GSEA implementation in J-Express 2012. As the Rank Product metric is intrinsically incompatible with the default weighted scoring scheme of GSEA, we opted for a log-fold scoring metric for evaluating gene-sets on our paired samples. This is the most comparable metric to the one used by the Rank Product method when sorting logratios of paired samples before combining them into a Rank Product. Other parameters were used with default settings: permutation method: genes, min number of members: 10, max number of members: 500.

### Trend Descriptions Based on Gene Ontology

Each dataset was analyzed independently by Rank Product, GO over-representation analysis and GSEA. The same procedure was first performed with focus on upregulation in invasive samples over angiogenic samples, then with focus on upregulation in angiogenic samples over invasive samples. The GO Terms and gene annotations of the top lists were manually screened for terms functionally relevant to angiogenesis and invasion, and the top list trends summarized from this.

### Mapping of Transcript and Protein Identifiers between Datasets

The human Entrez Gene ID for the targeted genes on the ABI microarray was used as the common identifier between the transcriptomics and proteomics data sets. Using the online ID converter service at BioMart Central Portal (http://central.biomart.org), the identified protein SwissProt IDs from the proteomics dataset was first mapped to their corresponding human or rat Entrez Gene IDs. The rat Entrez Gene IDs for the proteins identified as of host origin, were further mapped to the human Entrez Gene IDs for their homologous genes using BioMart’s gene retrieval service, with Ensembl transcript IDs as the linking identifier.

**Figure 2:**
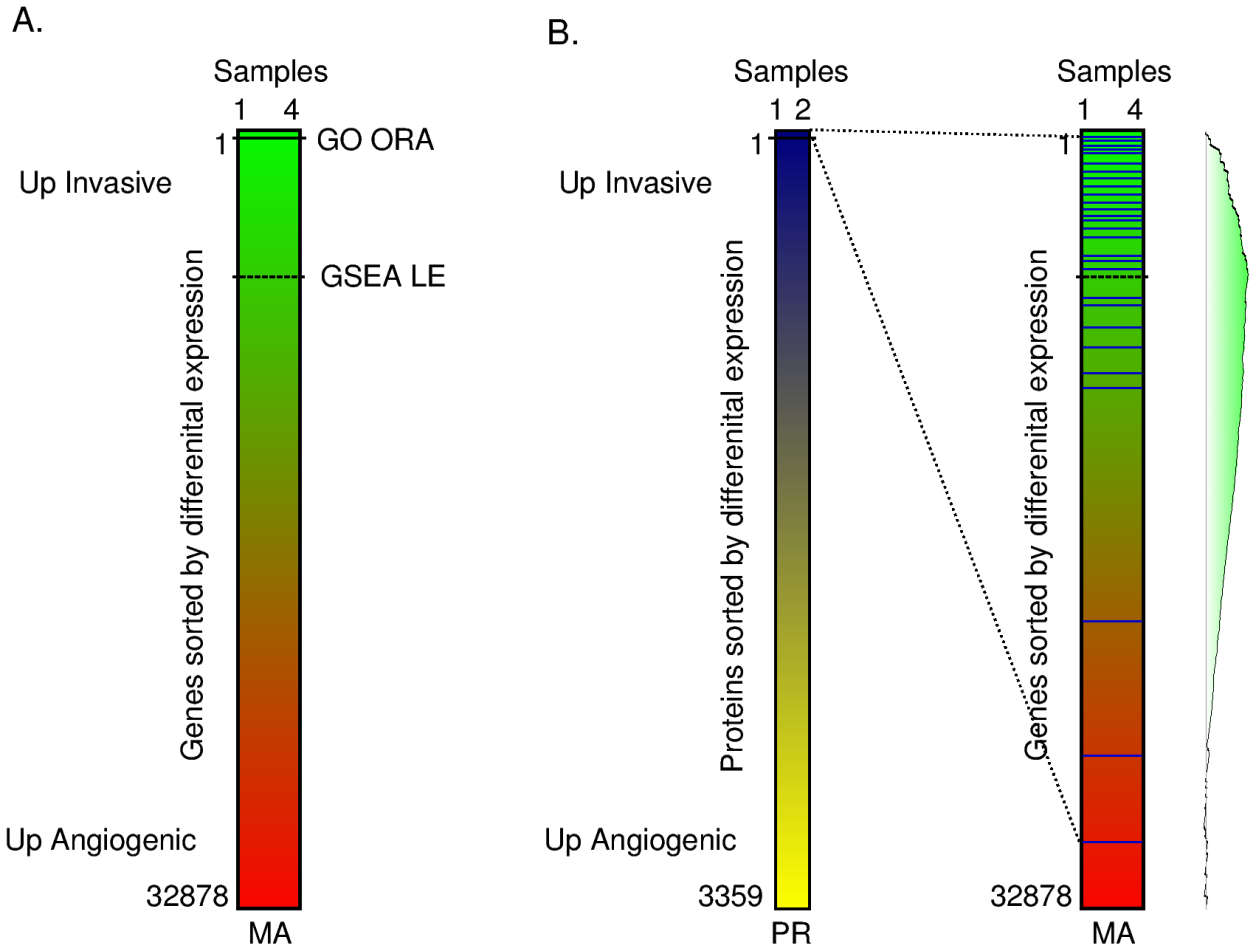
A: The datasets were analysed for differential expression independently using Rank Product, Gene Ontology over-representation (GO ORA) and GSEA. The methods evaluate different fractions of the datasets as biologically relevant when sorted for differential expression, as illustrated for the transcriptomics data set (TR). RP and GO ORA in our case only identified the top ∼1% of the overall sorted gene list as relevant, both for the transcriptomics and proteomics analysis. GSEA on the other hand identified Leading Edge (LE) subsets spanning ∼20% of the overall gene list. B: GSEA based approach for integrating partially overlapping proteomics and transcriptomics data sets. The top differentially expressed entities from one dataset is mapped into corresponding entities from the other dataset and evaluated as a gene set in GSEA. PR: Proteomics dataset, TR: Transcriptomics dataset.

After completing the mapping, it was then possible to analyze the transcripts corresponding to the top differentially expressed proteins as a gene set in the transcriptomics data, as illustrated in Figure 2B. The blue horizontal bars represent protein corresponding transcripts and how they distribute in the microarray data. The same analysis is done reversely for transcript corresponding proteins in the proteomics data.

### Public Availability of Data

The microarray data have been annotated according to MIAME [19] and are deposited in ArrayExpress (http://www.ebi.ac.uk/arrayexpress), accession no E-MTAB-1185. The normalized data matrix for the quantitative proteomics data is available in File S2.

### Rank Product Meta-analysis

The matching subsets of proteins and transcripts from the microarray and proteomics datasets were first identified. Then they were ranked individually according to differential expression between the invasive and angiogenic samples using the Rank Product (RP) statistics [13]. The resulting ranks were then used as the input to RP in a second meta-analysis step to identify protein-transcript pairs highly ranked in both individual analyses.

### Independent Microarray and Proteomics Dataset Validation Analysis

The CIA approach [15] discussed their method’s performance on the published mixorarray and proteomics data available for the life cycle of *Plasmodium falciparum,* a malaria parazyte [20]. We used the same published data sets, available as Tables S1 and S2 in File S1 from their publication, and log2 transformed the linear expression values for both datasets before proceeding with GSEA analysis. The datasets contain 4 consecutive asexual lifestages: merozoite, ring, trophozoite and schizout. We made a rough definition of expressed transcripts in a lifestage as the transcripts having a minimum expression value of 1000, yielding gene sets in the size range of 97–203, and for proteins, a minimum expression value of 50, yielding gene sets in the size range of 10–77 (gene sets are listed in File S3). The transcript based gene sets were analyzed for enrichment in all 4 life stages in the proteomics data using GSEA in J-Express (single class, weighted logfold scoring), and the protein based gene sets similarly in the microarray data.

## Results

### Analysis Results on Individual Data Sets

Table 1 summaries the results of the individual analysis, elucidating the trends that can be found in the brain cancer model proteomics and transcriptomics data sets individually using traditional analysis methods in combination with the Gene Ontology (www.geneontology.org, [18]). Figure 2A illustrates the proportions of the total gene lists that the different methods report findings from.

**Table 1:** Overview of trends and statistical significance from the individual analyses of the proteomics and transcriptomics datasets.

Analysis (Data set)	Invasive phenotype - early generation		Angiogenic phenotype - late generation	
	Trend description	Stat sign, list length	Trend description	Stat sign, list length
Rank Product (PR)	No trends observed (Table S1 in File S1)	*q* = 30%, 11 proteins (*q* = 50%, 28 proteins)	Some proteins related to angiogenesis (Table S2 in File S1)	*q* = 30%, 59 proteins (*q* = 50%, 132 proteins)
Rank Product (TR)	No trends observed (Table S3 in File S1)	*q* = 30%, 272 genes (*q* = 50%, 655 genes)	No trends observed (Table S4 in File S1)	*q* = 30%, 23 genes (*q* = 50%, 170 genes)
GO Over-representation (PR)	No trends observed (Table S5 in File S1)	*p*<0.02, 44 GO gene sets	Some proteins indicating late/angiogenic phenotype (Table S6 in File S1)	*p*<0.02, 75 GO gene sets
GO Over-representation (TR)	Central nervous system development and processes (Table S7 in File S1)	*p*<0.02, 298 GO gene sets	No trends observed (Table S8 in File S1)	*p*<0.02, 102 GO gene sets
GO GSEA (PR)	Neural development and regulation (Table S9 in File S1)	FDR = 50%, 7 GO gene sets	Increase in mitochondrial related gene sets (Table S10 in File S1)	FDR = 50%, 20 GO gene sets
GO GSEA (TR)	Neuron system development and regulation, biosynthetic processes (Table S11 in File S1)	FDR = 50%, 630 GO gene sets	Cell Cycle, Growth and Proliferation signature, blood vessel development (Table S12 in File S1)	FDR = 50%, 743 GO gene sets

PR: Proteomics, TR: Transcriptomics, GO: Gene Ontology, GSEA: Gene Set Enrichment Analysis, FDR: False Discovery Rate.

Although there are several GO terms/trends found overlapping between the individual proteomics and transcriptomics results, they seem to be highlighting some general terms for the angiogenic tumors. For the invasive phenotype there is more consistency in GO terms overlapping between proteomics and microarray results and the highlighted consensus trends of Table 1, than for the angiogenic type.

### Gene Set Based Integrated Data Analysis Approach

We suggest a new integrated analysis approach for the co-analysis of data sets with only a partial set of corresponding entities. By mapping the transcripts to the corresponding proteins (see M&M) we can assess how the top differentially expressed transcripts distribute as a set of proteins in the proteomics data, and how the top differentially expressed proteins distribute as a set of transcripts in the microarray data. See Figure 2B. We first identify the top up-regulated proteins using RP on the set of mapped proteins at a given significance level, both upregulated in invasive (I) and angiogenic (A), and screen the corresponding sets of transcripts using GSEA in the full microarray data set. Similarly we identify the top up-regulated transcripts using RP on the set of mapped transcripts at a given significance level, both in invasive and angiogenic samples, and screen the corresponding sets of proteins using GSEA in the full proteomics data set.

#### Microarray RP results support proteomics data in invasive samples

**Figure 3:**
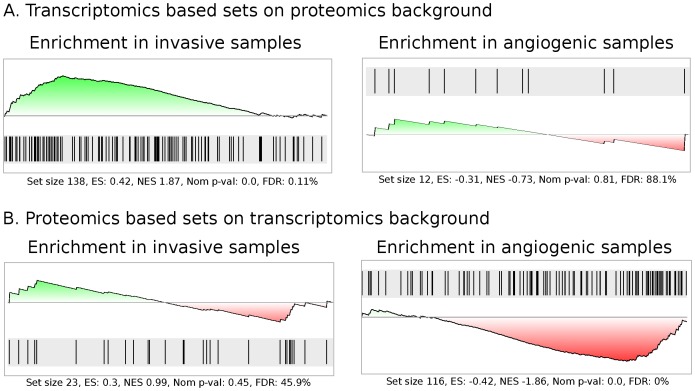
The results of the GSEA integrated analysis. A: left – transcript corresponding proteins enriched in invasive samples, right – transcript corresponding proteins enriched in angiogenic samples. B: left – protein corresponding transcripts enriched in invasive samples, right – protein corresponding proteins enriched in angiogenic samples.

As seen in Figure 3A, left panel, there is a significant enrichment in the proteomics data of the proteins corresponding to the differentially expressed transcripts up-regulated in the microarray data. The enrichment in invasive samples is consistent with the up-regulation of transcripts in invasive samples in the microarray data. The right panel shows for comparison, that there is no such significant trend for proteins corresponding to the transcripts up-regulated in the angiogenic samples.

The leading edge consisting of 47 transcript/protein combinations from this gene set is displayed in Table 2, and represents the starting point of biological interpretation of this integrated co-analysis.

**Table 2:** The set of proteins supported by corresponding transcripts as differentially expressed and upregulated in the invasive samples.

Gene Symbol	Protein Name	Species origin	iTRAQ experiment	Mapped Human Entrez ID	No of unique peptides	Cell Comp	Biol Proc
MBP	Myelin basic protein S	Rat	iTRAQ2	4155	12	neuron projection, myelin sheath	nervous system development, transmission of nerve impulse
MAG	Myelin-associated glycoprotein precursor	–	iTRAQ1	4099	9		nervous system development
NFL	Neurofilament triplet L protein	–	iTRAQ1	4747	16	neuron projection	nervous system development, transmission of nerve impulse
MBP	Myelin basic protein S (MBP S)	–	iTRAQ1	4155	23	neuron projection, myelin sheath	nervous system development
CROCC	Rootletin	–	iTRAQ1	9696	2		
MYPR	Myelin proteolipid protein	–	iTRAQ1	5354	10		
NFM	Neurofilament triplet M protein	Rat	iTRAQ1	4741	17	neuromuscular junction, neuron projection	nervous system development
BCAS1	Breast carcinoma amplified sequence 1 homolog	–	iTRAQ1	8537	8		
BASP	Brain acid soluble protein 1	Rat	iTRAQ2	10409	16		
GPR37	Probable G-protein coupled receptor 37 precursor	Rat	iTRAQ1	2861	5		
NFM	Neurofilament triplet M protein	Rat	iTRAQ2	4741	23	neuromuscular junction, neuron projection	nervous system development
MYPR	Myelin proteolipid protein	Human	iTRAQ2	5354	7		
PACN1	Protein kinase C and casein kinase substrate in neurons protein 1	–	iTRAQ2	29993	7		
NFL	Neurofilament triplet L protein	–	iTRAQ2	4747	22	neuron projection	nervous system development
NFASC	Neurofascin precursor	Rat	iTRAQ2	23114	15	neuron projection	nervous system development, transmission of nerve impulse
NDRG2	Protein NDRG2	–	iTRAQ1	57447	4		nervous system development
GBG3	Guanine nucleotide-binding protein	–	iTRAQ2	2785	3		
PRAX	Periaxin	–	iTRAQ2	57716	6		
NDRG2	Protein NDRG2	–	iTRAQ2	57447	3		nervous system development
AT2B3	Plasma membrane calcium-transporting ATPase 3	–	iTRAQ2	492	3		
NEGR1	Neuronal growth regulator 1 precursor	–	iTRAQ2	257194	3	neuron projection	
MAG	Myelin-associated glycoprotein precursor	–	iTRAQ2	4099	3		nervous system development
ITPR1	Inositol 1.4.5-trisphosphate receptor type 1	–	iTRAQ2	3708	2	postsynaptic density	
BSN	Protein bassoon	Rat	iTRAQ2	8927	84	presynaptic active zone	transmission of nerve impulse
PDK1	Pyruvate dehydrogenase lipoamide] kinase 1	–	iTRAQ1	5163	4		transmission of nerve impulse
GNAO1	Guanine nucleotide-binding protein G(o) subunit alpha 1	–	iTRAQ2	2775	18		nervous system development
ITPR1	Inositol 1.4.5-trisphosphate receptor type 1	Rat	iTRAQ1	3708	2		
KCC2B	Calcium/calmodulin-dependent protein kinase type II beta	Rat	iTRAQ2	816	14		
NFH	Neurofilament triplet H protein	Rat	iTRAQ2	4744	26	neuron projection	nervous system development
SNP25	Synaptosomal-associated protein 25	Human	iTRAQ2	6616	11		
SEPT4	Septin-4	–	iTRAQ1	5414	6		
NFH	Neurofilament triplet H protein	Rat	iTRAQ1	4744	20	neuron projection	nervous system development
SPTN2	Spectrin beta chain. brain 2	Rat	iTRAQ2	6712	45		
SYUA	Alpha-synuclein	–	iTRAQ2	6622	2		
KCC2B	Calcium/calmodulin-dependent protein kinase type II beta chain	–	iTRAQ1	816	13		
SYT2	Synaptotagmin-2	–	iTRAQ2	127833	4	synaptic vesicle mebrane	neurotransmitter secretion
LGI1	Leucine-rich glioma-inactivated protein 1 precursor	–	iTRAQ2	9211	6	synapse,	
AT2B1	Plasma membrane calcium-transporting ATPase 1	–	iTRAQ2	490	30		
CCG3	Voltage-dependent calcium channel gamma-3 subunit	–	iTRAQ2	10368	2		
PP2BA	Serine/threonine-protein phosphatase 2B catalytic subunit alpha isoform	–	iTRAQ2	5530	10		
CAMKV	CaM kinase-like vesicle-associated protein (1G5)	–	iTRAQ2	79012	7		
ALDOC	Fructose-bisphosphate aldolase C	Rat	iTRAQ2	230	12	neuron projection	
NEGR1	Neuronal growth regulator 1 precursor	–	iTRAQ1	257194	2	neuron projection	
BASP	Brain acid soluble protein 1	Rat	iTRAQ1	10409	16		
PCD10	Protocadherin-10 precursor	–	iTRAQ2	57575	2		
SYN2	Synapsin-2	Rat	iTRAQ2	6854	16	synaptic vesicle	transmission of nerve impulse, neurotransmitter secretion
VISL1	Visinin-like protein 1	Human	iTRAQ2	7447	5		

Table 2 consist of the 47 protein profiles in the leading edge of the gene set found to be clearly enriched in the invasive samples (Figure 3). The gene set was defined as the set of proteins matching the transcripts found differentially expressed in the Rank Product analysis of the transcriptomics dataset. Signal log2 ratios are listed in Table S13 in File S1. Cell Comp = Gene Ontology cellular component annotations. Biol Proc = GO biological process annotations. The same protein (Gene Symbol/Entrez ID) may appear several times since it can be identified from different origins and/or in different independent iTRAQ experiments.

#### Proteomics RP results support microarray data in angiogenic samples

Figure 3B, right panel, displays the significant enrichment in the microarray data of the transcripts corresponding to the differentially expressed proteins in the proteomics data. The enrichment in angiogenic samples is consistent with the up-regulation of proteins in angiogenic samples in the proteomics data. The left panel shows for comparison that there is no significant trend for transcripts corresponding to proteins up-regulated in the invasive samples.

The leading edge consisting of 43 transcripts backed up by protein data, is listed in Table 3, and plain inspection of the list reveals many genes previously found related to angiogenesis.

**Table 3:** The set of genes supported by corresponding proteins as differentially expressed and upregulated in the angiogenic samples.

Probe ID	Gene Symbol	Gene Name	Human Entrez Gene ID	Cell Comp	Biol Proc
167807	A2M	alpha-2-macroglobulin	2		
226560	CALU	calumenin	813		blood coagulation, platelet activation
213714	VAV3	vav 3 oncogene	10451	cell periphery	developmental process, angiogenesis, blood coaculation, platelet activation
185731	CALD1	caldesmon 1	800	cell periphery	
164486	KIAA0152	KIAA0152	9761	membrane	
209540	ANXA2P2	annexin A2 pseudogene 2	302	cell periphery	developmental process, angiogenesis
162321	SCARB2	scavenger receptor class B, member 2	950	membrane, cell periphery	
158386	EPN2	epsin 2	22905		
175324	GAPDH	glyceraldehyde-3-phosphate dehydrogenase	2597	membrane	
127943	PRKCSH	protein kinase C substrate 80K-H	5589		
203857	GANAB	glucosidase, alpha	23193		
186473	SLC16A1	solute carrier family 16 (monocarboxylic acid transporters), member 1 (MCT1)	6566	membrane, cell periphery	blood coagulation
184288	CALU	calumenin	813		
211060	–	unassigned	483		
100826	ANXA2	annexin A2	302	cell periphery	developmental process, angiogenesis
218154	–	unassigned	483		
125960	CRIP2	cysteine-rich protein 2	1397		
235868	–	unassigned	2597		
184373	PDIA4	protein disulfide isomerase family A, member 4	9601		
207626	ANXA2	annexin A2	302	cell periphery	developmental process, angiogenesis
217860	KLC3	kinesin light chain 3	147700		
168192	DNAJB11	DnaJ (Hsp40) homolog, subfamily B, member 11	51726		
178526	PDIA6	protein disulfide isomerase family A, member 6	10130	cell periphery	
137362	CANX	calnexin	821	membrane	developmental process
122012	PDIA6	protein disulfide isomerase family A, member 6	10130	cell periphery	
211546	P4HB	procollagen-proline, 2-oxoglutarate 4-dioxygenase (proline 4-hydroxylase), beta polypeptide (protein disulfide isomerase-associated 1)	5034	cell periphery	
163592	YBX1	Y box binding protein 1	4904		
135268	KIAA1271	KIAA1271 protein	57506	membrane	
201478	MCCC2	methylcrotonoyl-Coenzyme A carboxylase 2 (beta)	64087		
119206	SCARB2	scavenger receptor class B, member 2	950	membrane, cell periphery	
197538	BHLHB2	basic helix-loop-helix domain containing, class B, 2	8553		
104131	PDIA3	protein disulfide isomerase family A, member 3	2923		
139743	C1orf43	chromosome 1 open reading frame 43	25912	membrane	
182757	–	unassigned	4904		
214214	ANXA5	annexin A5	308		blood coagulation
129188	MSN	moesin	4478	membrane, cell periphery	
144383	–	unassigned	4904		
174598	–	unassigned	7726		
147730	RBM19	RNA binding motif protein 19	9904		developmental process
139584	HLA	major histocompatibility complex, class I, A	3105		
119283	PYCR1	pyrroline-5-carboxylate reductase 1	5831		
134467	CKAP4	cytoskeleton-associated protein 4	10970	membrane	
136724	TMEM16E	transmembrane protein 16E	203859	membrane	

Table 3 consist of the 43 transcript measurements in the leading edge of the gene set found to be clearly enriched in the angiogenic samples (Figure 3). The gene set was defined as the set of transcripts matching the the proteins found differentially expressed in the Rank Product analysis of the proteomics dataset. Signal log2 ratios are listed in Table S14 in File S1.

### Comparison to Standard Method and Independent Data Validation

A straightforward meta-analysis of the brain cancer model microarray and proteomics datasets revealed no significant corresponding transcript and protein pairs being differentially expressed between invasive and angiogenic samples. (Invasive vs angiogenic top 20 pairs, *q* = 83.9%, angiogenic vs invasive top 20 pairs, *q* = 78.1%, see File S4).

The GSEA results from evaluating the top expressed proteins in the different life cycle stages of *Plasmodium falciparum* against the transcriptomics datasets for the same life cycle stages are collected and presented in File S3. Likewise are the results for the top expressed transcripts analyzed against the proteomics datasets of the different stages. These are contrasted with the results in Table 2 of the original work [20].

## Discussion

Gene-set based methods often elude more than straightforward gene-by-gene differential expression analysis, and have received some focus in the recent years. Another alternative to strengthen the statistical power within an experiment; say a microarray experiment, through adding more samples (replicates) for the statistical test to compute from, is to combine results from several independent experiments, that together display a trend as significant. Sometimes this is referred to as a meta-analysis, depending on level of abstraction from the original data, and sometimes as an integrated approach. Common to both is the necessity to map entities from different datasets to each other and the use of a suitable statistical test to evaluate the combined model. As demonstrated for the brain cancer model datasets, a regular Rank Product meta-analysis fails in this case to identify significant support between the datasets, and alternative ways of relating the datasets in an integrated approach is called for.

As seen in Table 1, the different traditional analysis approaches have difficulties finding truly statistically significant results on their own. The trends discovered are meaningful in terms of the general difference between the invasive and angiogenic phenotype, but are neither very specific nor associated with convincing confidence levels.

Based on the results of manual analyses (resumed in Table 1) we can conclude that the invasive type of the experimental tumors is connected with Gene Ontology terms indicating sets of genes involved in central nervous system development, it’s processes and regulation, as assessed by GO over-representation analysis in the transcriptomics data and by the GSEA approach in both the proteomics and transcriptomics data. This is in agreement with the phenotypic appearance and behavior of invasive tumors, which resemble a more immature stem-like cell, capable to infiltrate neighboring structures, much like neural stem cells do in the developing brain. The angiogenic phenotype however, is connected with the genes related to angiogenesis as assessed by RP analysis, GO over-representation analysis in proteomics as well as GSEA in transcriptomics which also included the representation of terms linked to cell cycle, growth and proliferation.

In contrast to the analyses of the individual datasets, the integrated analysis shows two important statistically significant trends: 1) up-regulated transcripts in the invasive phenotype evaluated together is found as a set of proteins significantly up-regulated together in the invasive phenotype, 2) up-regulated proteins in the angiogenic phenotype evaluated together is found as a set of transcripts significantly up-regulated together in the angiogenic phenotype. As Figure 3 shows, the leading edges of these sets are spanning roughly 20% of the full background list. Hence our co-analysis approach identifies significant gene sets in the same background gene lists all the individual analyses in Table 1 were evaluating.

A strong consistency between the integrated analysis results in Table 2 and the weaker individual analysis results from Table 1 is confirmed by plain inspection of the protein names in Table 2 and the dominance of neuronal development and activity related descriptions. In addition we have listed the most relevant Gene Ontology terms the 47 proteins in Table 2 are annotated with, and these are clearly matching the scope of the terms identified by individual analyses (in particular Table S9 in File S1). In the case of the invasive phenotype of this experimental GBM model the tumor cell (human) infiltration of the host (rat) brain tissue is so vast that it is virtually impossible to isolate or surgically remove the pure tumor by surgical means, which is also one of the major issues in the poor success of the surgical treatment alone for human GBMs. Therefore the tumor tissue samples of this phenotype are ‘contaminated’ to a large extent by host (rat) brain tissue. The proteins identified by the integrated analysis as differentially expressed as a set, upregulated in the invasive phenotype, as well as the results of GSEA of proteomics (Table S9 in File S1) and manual cross comparisons using the Ingenuity Pathway Analysis and Human Protein Atlas confirmed this situation at the level of proteins. Almost half (17 of 36 unique proteins – Table 2) are in fact proteins linked to the brain cellular localization (cellular component) and are either of neural (Synapse, Neuromuscular junction, Postsynaptic density, Synaptic vesicle, Presynaptic vesicle membrane, Presynaptic active zone, Neuronal cell body etc.) or glial origin (Myelin sheath, Compact myelin, etc.) and mostly host proteins or sharing protein sequence homology with the host.

In addition both the GO over-representation analysis (Table S7 in File S1) and GSEA (Table S11 in File S1) of the transcriptomics data are strongly dominated by brain related terms indicating host origin rather than tumor cells.

Table 3 lists the up-regulated set of transcripts in the angiogenic samples which are supported by the proteomics data, the most dominant trend overlapping with the individual analysis results are developmental process and blood vessel formation. In particular the presence of the concrete term angiogenesis annotating three genes (vav3, anxa2 and anxa2p2) in Table 3 is very interesting. This is the first time we by molecular level assays were able to indicate the term reflecting *de facto* angiogenesis in late generation tumors (Figure 1), as being one of the most important phenotypic characteristics of the late generation glioma animal model as well as one of the hallmarks of the high grade glioma in patient. Moreover the expression of anxa2 was thoroughly validated at the level of immunohistochemistry in additional tissue samples of GBM xenograft models as well as on large number of more than 200 clinical gliomas samples of various grades in a form of a tissue microarray as shown in our previous research. Indeed we confirmed a strong up-regulation of Anxa2 in angiogenic xenografts compared to invasive ones, as well as a significant increase in Anxa2 expression in high grade gliomas (grade III and IV) compared to low grades (grade I and II) [9].

The over-representation of membrane localized proteins (plasma membrane, ER, GA and in some instances the Mt) seen in Table 3, can be explained by the experimental setup of the proteomics experiment which included an enrichment step for membrane proteins. Hence the integrated analysis also will have a bias towards transcripts with gene products in these cellular compartments. This may also explain the fact that we do not see support for the signature of cell cycle, growth and proliferation that was seen as a major trend in the individual analyses (Table S12 in File S1 in particular). Upon closer examination of the cellular localization of the underlying transcripts for the trend in Table S12 in File S1, a majority of these were annotated as located in the nucleus, and corresponding proteins will thus less likely be picked up in the membrane targeted fraction in the proteomics experiment.

Individual analyses pointing towards cell adhesion terms (Table S11 in File S1) are supported by the integrated approach (Table 3, MSN) and are in accordance with the invasive phenotype where cell adhesion appears to may be disrupted.

In addition to providing support for the different individual analyses and contributing to a more holistic interpretation of the two datasets, the integrated analysis results in a focused list of transcripts and proteins found up-regulated in the two phenotypes for further analysis. It is clear from Table 3 that many of these have previously been identified as related to angiogenesis and that this set in combination with the other results is an excellent starting point for further investigation of this brain cancer model.

As an independent test of the integrated approach to identify support of a trend in one dataset from another dataset on related samples, we tried to rediscover a main result of Le Roch et al [20]. Between the four asexual life cycle stages of *Plasmodium falciparum* in their data, they identified a stronger relationship between the trancriptomics expression level in one stage and the protein expression levels in the following life cycle stage, than directly within in the same stage. For protein gene sets evaluated in the microarray data using GSEA, we find the next life cycle stage as the most enriched protein gene-set in three out of three transitions, compared to two out of three in the original work. For the reversed direction, the previous life cycle stage transcript gene-set was among the two top ranked sets in all three transitions, two times out-competed by the corresponding life cycle stage at rank 1. These results emphasize the potential of the integrated approach to detect and evaluate relationships between multiple datasets on related samples.

Future work should include a follow up on the focused lists of Tables 2 and 3, as well as the individual analyses results strengthened by the integrated analyses, in the context of functional experiments work in the brain cancer model. In addition since the integrated results focused on overlapping entities only between the proteomics and transcriptomics datasets, a study of how the core set of overlapping proteins and transcripts can be extended based on functional annotation is warranted.

## Supporting Information

Files S1 **Tables S1–Table S14.** Table S1 in File S1 - Rank Product analysis in proteomics dataset, enriched in invasive phenotype (early generation). Table S2 in File S1 - Rank Product analysis in proteomics dataset, enriched in angiogenic phenotype (late generation). Table S3 in File S1 - Rank Product analysis in transcriptomics dataset, enriched in invasive phenotype (early generation). Table S4 in File S1 - Rank Product analysis in transcriptomics dataset, enriched in angiogenic phenotype (late generation). Table S5 in File S1 - GO Overrepresentation analysis in proteomics dataset, enriched in invasive phenotype (early generation). Table S6 in File S1 - GO Overrepresentation analysis in proteomics dataset, enriched in angiogenic phenotype (late generation). Table S7 in File S1 - GO Overrepresentation analysis in transcriptomics dataset, enriched in invasive phenotype (early generation). Table S8 in File S1 - GO Overrepresentation analysis in transcriptomics dataset, enriched in angiogenic phenotype (late generation). Table S9 in File S1 - GO GSEA in proteomics dataset, enriched in invasive phenotype (early generation). Table S10 in File S1 - GO GSEA in proteomics dataset, enriched in angiogenic phenotype (late generation). Table S11 in File S1 - GO GSEA in transcriptomics dataset, enriched in invasive phenotype (early generation). Table S12 in File S1 - GO GSEA in transcriptomics dataset, enriched in angiogenic phenotype (late generation). Table S13 in File S1– Expression signal log2 ratios for identified proteins in Table 2. Table S14 in File S1– Expression signal log2 ratios for identified transcripts in Table 3.
(XLS)

File S2 **Preprocessed and normalized Rat Brain Model proteomics data set.**
(TXT)

File S3 **Gene sets and results for the integrated approach applied to an independent** ***Plasmodium falciparum*** **pair of proteomics and transcriptomics data sets.**
(XLS)

File S4 **Rank Product meta-analysis results of proteomics and transcriptomics data for the Rat Brain Model samples.**
(XLS)
